# Complementary and alternative medicine use amongst patients with cardiovascular disease in Singapore

**DOI:** 10.1186/s12906-016-1430-4

**Published:** 2016-11-08

**Authors:** Tse Yean Teo, Jonathan Yap, Tong Shen, Khung Keong Yeo

**Affiliations:** 1 Yong Loo Lin School of Medicine, National University of Singapore, Singapore, Singapore; 2 National Heart Center Singapore, 5 Hospital Drive, Singapore, 169609 Singapore; 3 Duke-NUS Graduate Medical School, Singapore, Singapore

**Keywords:** Complementary and alternative medicine, Cardiovascular disease, Singapore, Communication

## Abstract

**Background:**

Data on complementary and alternative medicine (CAM) use in patients with cardiovascular disease (CVD) are lacking. We aim to investigate the prevalence of CAM use among patients with CVD attending a tertiary centre for cardiovascular care, their attitudes and beliefs towards CAM, and factors associated with CAM usage.

**Methods:**

A cross-sectional, self-administered written survey was conducted on consecutive patients attending outpatient cardiovascular clinics at our tertiary institution over 2 months from June to July 2014. Information gathered included demographic data and various aspects of CAM use.

**Results:**

A total of 768 responses (562 males, mean age 57 ± 13 years, 74 % Chinese, 6 % Malay, 14 % Indian) were included. The prevalence of CAM use in the cohort was 43.4 % (333/768). Biologically-based systems (29.4 %) was the most common type of CAM used. Some patients (19.0 %) used multiple types of CAM simultaneously. External influences (78.1 %) were cited more than internal influences (47.8 %) to affect CAM use. Malay ethnicity (compared to Chinese) was the only significant negative multivariate predictor of CAM use (OR = 0.531 (95 % CI 0.147 to 0.838), *p* = 0.018). A significantly higher proportion of CAM users compared to non-CAM users were non-compliant to medications (35.6 %, *n* = 114 vs. 20.5 %, *n* = 84, *p* < 0.001) and consults (41.4 %, *n* = 130 vs. 28.1 %, *n* = 112, *p* < 0.001) respectively.

**Conclusion:**

The usage of CAM is prevalent amongst our patients with CVD. CAM use was associated with poorer reported compliance to medications and consults. Understanding the factors influencing CAM use amongst CVD patients provides medical professionals with an opportunity to better discuss CAM use and potentially enhance the patient-physician interaction.

**Electronic supplementary material:**

The online version of this article (doi:10.1186/s12906-016-1430-4) contains supplementary material, which is available to authorized users.

## Background

Complementary and alternative medicine (CAM) is commonly used for treatment of cardiovascular diseases (CVD) in many countries [1, 2]. CAM is defined as a group of diverse medical and healthcare systems, practices and products that are not presently considered to be part of conventional medicine [3]. Studies conducted in Western countries report as much as 65 % of the study population using some form of CAM [4–6]. In Singapore, CAM use amongst patients is prevalent [7, 8]. Past studies have evaluated CAM use in local cancer patients [8–10], diabetes [11] and epilepsy [12], but data in patients with CVD are lacking.

The usage of CAM could have potential implications for patients and doctors. Poor communication between patients and doctors regarding CAM use has been reported overseas [13, 14] and locally [7]. Patients were unaware of potential health risks of CAM [15], including drug interactions, side effects and non-compliance to conventional medicine [16]. This is especially important in patients with CVD, who may consume medications with narrow therapeutic index and extensive drug interaction profiles [17]. In addition, non-compliance to medications and visits have been shown to be a strong predictor of adverse outcomes in CVD patients [18–20]. As such, the use of CAM could result in poor patient outcomes [21] given that the potential benefits of CAM in CVD in general are not well established [22].

In this study, we aim to investigate the prevalence of CAM use among patients with CVD (including coronary artery disease, cardiac failure, stroke) attending a tertiary centre for cardiovascular care, their attitudes and beliefs towards CAM use, as well as factors associated with CAM usage.

## Methods

A cross-sectional, self-administered written survey was conducted on consecutive patients attending outpatient cardiovascular clinics at our tertiary institution over a period of 2 months from June to July 2014. Approval for the study was obtained from the SingHealth Centralised Institutional Review Board.

The questionnaire, available in English and Chinese, was developed based on similar topics used in previous studies [10, 15], and adapted to a local context. The questionnaire was pilot tested among twenty patients with similar profiles to our target population and further refined to ensure that all the questions were easily understood and interpreted uniformly based on feedback from the pilot. Responses were recorded using a 4 point Likert scale. Clinical information from the questionnaire was supplemented from patients’ hospital medical records. These included clinical characteristics and risk factors (eg. diabetes mellitus, hypertension, hyperlipidemia), physical examination findings (eg. Body mass index, systolic blood pressure and diastolic blood pressure), laboratory test results (eg. HbA1c, LDL) and medications. The presence of risk factors like diabetes, mellitus, hypertension and hyperlipidemia was based on the self-reported questionnaire and/or documentation of such a clinical condition from the records.

In the questionnaire, the term “non-western medicine” was used in lieu of CAM as it facilitated better understanding of the term in our local context based on data from the pilot study. As seen from question 17 of the questionnaire (see Additional file 1), the components are similar. We classified types of CAM into 4 main groups [3] namely (1) biologically-based systems (eg. Traditional Chinese Medicine (TCM), Jamu (Malay traditional medicine), herbs, vitamins), (2) manipulative and body-based methods (eg. massage, acupuncture, chiropractice), (3) mind-body interventions (eg. relaxation therapies like yoga and meditation, tai chi, chi gong) and (4) energy therapies (eg. magnetic therapy). CAM that were not specified by the participants were classified under (5) others. The data was analysed based on this grouping. Questions to ascertain the physician’s role in the patient’s use of CAM, self-reported compliance to conventional medicine, follow-up appointments and trust for doctors were also included. A sample of the questionnaire is included in the Additional file 1.

### Statistical analysis

Statistical analysis was performed using Statistical Package for the Social Sciences (SPSS) 22.0 for Windows (SPSS Inc, Chicago, IL). The demographic and risk factor profile of the study population was characterized using descriptive statistics. Comparison between the two groups (CAM users vs CAM non-users) was performed using Student’s *t*-test for parametric data, Mann-Whitney *U* test for nonparametric data and Chi-squared test for categorical data. The relationship between CAM usage and other factors was explored using bivariate correlational analyses and multivariate linear regression models.

## Results

The initial response rate was 71.1 % (1000 out of 1406 patients). Of the 1000 patients which responded, 768 had complete responses (562 males, mean age 57 ± 13 years, 74 % Chinese, 6 % Malay, 14 % Indian), the remaining were excluded due to incompleteness of response. See Table 1.

**Table 1:** Demographics of the study population

	Overall (*n* = 768)	CAM user (*n* = 333)	CAM non-user (*n* = 435)	*P*-value^a^
Demographics				
Age	56.8 (12.6)	56.8 (10.9)	56.7 (13.7)	0.963
Gender				0.442
Male	562 (73.2 %)	239 (71.8 %)	323 (74.3 %)	
Female	206 (26.8 %)	94 (28.2 %)	112 (25.7 %)	
Ethnicity				0.010
Chinese	569 (74.1 %)	264 (79.3 %)	305 (70.1 %)	
Malay	43 (5.6 %)	10 (3.0 %)	33 (7.6 %)	
Indian	107 (13.9 %)	39 (11.7 %)	68 (15.6 %)	
Others	49 (6.4 %)	20 (6.0 %)	29 (6.7 %)	
Education				0.485
≤ Secondary/ITE	243 (32.1 %)	112 (34.1 %)	131 (30.5 %)	
Polytechnic diploma/A Level	235 (31.0 %)	102 (31.1 %)	133 (31.0 %)	
Degree/Post-graduate	279 (36.9 %)	114 (34.8 %)	165 (38.5 %)	
Occupation				0.750
White collar	315 (51.6 %)	134 (53.4 %)	181 (50.4 %)	
Blue collar	101 (16.6 %)	41 (16.3 %)	60 (16.7 %)	
Unemployed/homemaker	194 (31.8 %)	76 (30.3 %)	118 (32.9 %)	
Housing				0.672
≤ 3 room public apartment	74 (10.4 %)	35 (11.5 %)	39 (9.6 %)	
4-5 room public apartment	350 (49.2 %)	146 (47.9 %)	204 (50.2 %)	
Private property	287 (40.4 %)	124 (40.7 %)	163 (40.1 %)	
Smoking				0.112
No	522 (71.7 %)	217 (68.7 %)	305 (74.0 %)	
Ever smoker	206 (28.3 %)	99 (31.3 %)	107 (26.0 %)	
Alcohol				0.933
No	337 (46.0 %)	144 (45.9 %)	193 (46.2 %)	
Ever drinker	395 (54.0 %)	170 (54.1 %)	225 (53.8 %)	
Clinical Characteristics				
BMI	25.7 (5.2)	25.7 (5.3)	25.7 (5.2)	0.899
Systolic Blood Pressure	131.4 (17.6)	132.2 (18.2)	130.8 (17.2)	0.284
Diastolic Blood Pressure	70.9 (9.8)	71.4 (10.0)	70.4 (9.7)	0.157
Diabetes mellitus	168 (21.9 %)	69 (20.7 %)	99 (22.8 %)	0.498
Hypertension	374 (48.7 %)	165 (49.5 %)	209 (48.0 %)	0.680
Hyperlipidemia	489 (61.4 %)	214 (64.3 %)	275 (63.2 %)	0.765
HbA1C (%)^b^	6.3 (1.4)	6.3 (1.4)	6.4 (1.3)	0.588
LDL Cholesterol (mmol/L)^c^	2.7 (1.0)	2.7 (1.0)	2.7 (1.0)	0.488

Abbreviations and definitions

*ITE* Institute of Technical Education. *HbA1C* Glycated hemoglobin, *White collar* workers perform job duties in an office setting, *Blue collar* workers perform labour jobs or work with their hands, *Public apartment* refers to heavily subsidized housing built by the government. Families with gross monthly income in excess of $10,000 are not eligible to directly purchase these subsidized apartments from the Housing and Development Board

Mean and SD are reported for continuous data and frequency and percentages for categorical data

^a^Comparing CAM users and CAM non-users

^b^HbA1C – 473 patients with missing data

^c^LDL – 303 patients with missing data

The prevalence of CAM use in the cohort was 43.4 % (333/768). Types of CAM use included biologically-based systems (29.4 %), manipulative/body based therapies (22.3 %), mind-body systems (11.3 %), energy therapies (1.2 %) and others (5.1 %). Some patients (19.0 %) used more than one type of CAM simultaneously. Among CAM users, concomitant usage of prescribed medications are as follows; beta-blockers (41.1 %), aspirin (37.5 %), angiotensin converting enzyme (ACE) inhibitors and/or angiotensin receptor blockers (ARB) (33.6 %), calcium channel blockers (26.1 %), clopidogrel (18.3 %), oral hypoglycemic agents (15.3 %), warfarin (1.8 %) and digoxin (1.8 %).

Since a significant proportion of CAM users (146/333) use more than one type of CAM, we compared patient characteristics between CAM users that use more than one type of CAM and CAM users that only use a certain type of CAM strictly, for which the top three analysed were biologically-based systems (27.3 %), manipulative/body based therapies (13.2 %) and mind-body systems (3.3 %). See Table 2.

**Table 2:** Patient characteristics based on types of CAM use

	Biologically-based systems (*n* = 91)	Manipulative and body-based methods (*n* = 44)	Mind-body interventions (*n* = 11)	More than 1 type of CAM used (*n* = 146)	*P*-value^a^
Demographics					
Age	57.3 (9.7)	57.6 (11.8)	57.2 (11.0)	56.6 (11.0)	0.867
Gender					0.262
Male	68 (74.7 %)	36 (81.8 %)	7 (63.6 %)	98 (67.1 %)	
Female	23 (25.3 %)	8 (18.2 %)	4 (36.4 %)	48 (32.9 %)	
Ethnicity					0.026
Chinese	72 (79.1 %)	35 (79.5 %)	6 (54.5 %)	118 (80.8 %)	
Malay	3 (3.3 %)	2 (4.5 %)	0 (0.0 %)	4 (2.7 %)	
Indian	8 (8.8 %)	2 (4.5 %)	4 (36.4 %)	20 (13.7 %)	
Others	8 (8.8 %)	5 (11.4 %)	1 (9.1 %)	4 (2.7 %)	
Education					0.136
≤ Secondary/ITE	38 (42.7 %)	15 (34.1 %)	0 (0.0 %)	39 (27.1 %)	
Polytechnic diploma/A Level	24 (27.0 %)	15 (34.1 %)	4 (40.0 %)	51 (35.4 %)	
Degree/Post-graduate	27 (30.3 %)	14 (31.8 %)	6 (60.0 %)	54 (37.5 %)	
Occupation					0.751
White collar	37 (54.4 %)	16 (47.1 %)	4 (80.0 %)	62 (55.4 %)	
Blue collar	11 (16.2 %)	8 (23.5 %)	0 (0.0 %)	14 (12.5 %)	
Unemployed/homemaker	20 (29.4 %)	10 (29.4 %)	1 (20.0 %)	36 (32.1 %)	
Housing					0.880
≤ 3 room public apartment	8 (9.6 %)	4 (10.0 %)	0 (0.0 %)	17 (12.7 %)	
4–5 room public apartment	40 (48.2 %)	22 (55.0 %)	4 (40.0 %)	63 (47.0 %)	
Private property	35 (42.2 %)	14 (35.0 %)	6 (60.0 %)	54 (40.3 %)	
Smoking					0.647
No	57 (67.1 %)	27 (64.3 %)	8 (72.7 %)	99 (72.3 %)	
Ever smoker	28 (32.9 %)	15 (35.7 %)	3 (27.3 %)	38 (27.7 %)	
Alcohol					0.709
No	35 (41.7 %)	15 (38.5 %)	6 (54.5 %)	68 (47.9 %)	
Ever drinker	49 (58.3 %)	24 (61.5 %)	5 (45.5 %)	74 (52.1 %)	
Clinical Characteristics					
BMI	25.5 (5.6)	26.3 (5.1)	24.3 (2.3)	25.8 (5.3)	0.906
Systolic Blood Pressure	130.4 (20.1)	134.0 (17.0)	122.1 (17.1)	132.3 (17.6)	0.484
Diastolic Blood Pressure	69.8 (9.5)	73.7 (11.3)	69.1 (10.2)	71.2 (9.9)	0.606
Diabetes mellitus	30 (33.0 %)	9 (20.5 %)	1 (9.1 %)	27 (18.5 %)	0.068
Hypertension	39 (42.9 %)	27 (61.4 %)	2 (18.2 %)	77 (52.7 %)	0.057
Hyperlipidemia	55 (60.4 %)	28 (63.6 %)	6 (54.5 %)	99 (67.8 %)	0.749
HbA1C (%)^b^	6.7 (2.0)	6.1 (0.8)	5.6 (0.2)	6.2 (1.3)	0.279
LDL Cholesterol (mmol/L)^c^	2.7 (1.0)	2.6 (1.0)	2.4 (0.6)	2.7 (0.9)	0.950

Energy therapies not included in analysis as only one patient used energy therapy only

Mean and SD are reported for continuous data and frequency and percentages for categorical data

^a^Comparing one type of CAM use only (excluding energy therapies and others) and more than one type of CAM use

^b^HbA1C – 473 patients with missing data

^c^LDL – 303 patients with missing data

Reasons for using CAM was classified into external influences (78.1 %), internal influences (47.8 %) and others (6.6 %). External influences come from friends and family (62.5 %), recommendation from the doctor (20.1 %) and media (8.4 %). Internal influences included perceiving CAM as having less side effects (36.3 %), better efficacy of CAM (10.2 %), lower cost of CAM (7.5 %), and poor results from conventional western medicine (6.6 %). (Table 3) Malay ethnicity (as compared to the Chinese) was the only significant negative multivariate predictor of CAM use (OR = 0.531 (95 % CI 0.147 to 0.838), *p* = 0.018) (Table 4).

**Table 3:** Reasons for CAM usage

	CAM users (*n* = 333)
External influences	260 (78.1 %)
Family/friends recommendation	208 (62.5 %)
Doctor’s recommendation	67 (20.1 %)
Media influence	28 (8.4 %)
Internal influences	159 (47.8 %)
Poor results from Western medicine	22 (6.6 %)
CAM is cheaper	25 (7.5 %)
CAM works better	34 (10.2 %)
CAM has less side effects	21 (36.3 %)
Others	22 (6.6 %)

Percentages are reported for categorical data

**Table 4:** Multivariate predictors of CAM use

	Prevalence ratio	95 % CI for prevalence ratio	*p*-value
Sex			
Male	0.880	0.597 to 1.299	0.521
Ethnicity			
Others	0.622	0.305 to 1.267	0.191
Indian	0.851	0.510 to 1.421	0.537
Malay	0.351	0.147 to 0.838	0.018
Chinese*			
Age	0.995	0.979 to 1.011	0.535
Education			
Diploma/A Level	0.974	0.625 to 1.518	0.909
Degree/Postgraduate	0.678	0.426 to 1.079	0.101
< Secondary school/ITE*			
Occupation			
Blue collar	1.157	0.654 to 2.047	0.616
White collar	1.218	0.787 to 1.884	0.376
Retired/unemployed*			
Housing			
≤ 3 room public flat	0.979	0.517 to 1.852	0.947
4–5 room public flat	0.835	0.561 to 1.243	0.375
Private Property*			

*Reference group

Approximately half of patients (50.8 %, *n* = 169) found CAM effective in treating their heart condition, while a smaller proportion (25.2 %, *n* = 84) found CAM more effective than conventional western medicine in treating their heart condition. Perceived effectiveness was highest in patients who used biologically based systems (31.5 %) and lowest in energy therapies (1.2 %). A small number of CAM users (7.8 %, *n* = 26) experienced side effects from CAM which included gastrointestinal complaints, general malaise, allergy and rash.

A significantly higher proportion of CAM users compared to non-CAM users (35.6 %, *n* = 114 vs. 20.5 %, *n* = 84, *p* < 0.001) were non-compliant (forgot to take medicines occasionally or most of the time) to medications. More CAM users reported non-compliance to doctor visits compared to non-CAM users (41.4 %, *n* = 130 vs. 28.1 %, *n* = 112, *p* < 0.001). Most patients reported that their doctors did not ask them about their usage of CAM (60.5 %, *n* = 465). Among CAM users, only 66 (19.8 %) asked their doctors about their CAM usage; reasons include – their doctor did not ask them first (37.2 %, *n* = 124), lack of time during the consult (11.4 %, *n* = 38), doctor would disapprove (8.4 %, *n* = 28) and doctor would not understand (5.4 %, *n* = 18). A sizable proportion (72.8 %, *n* = 559) would like to know more about CAM from their doctors.

## Discussion

The use of CAM in CVD patients in Western countries is prevalent ranging from 36–64 % [15, 23–25]. In Singapore, there is a lack of data on CAM use in CVD patients. However, in cancer patients, the use of CAM was found to be at least half at about 55–56 % [8, 9]. In our cohort, we found similar rates of CAM use at about 43.4 %.

Out of the total CAM users, a significant number (43.8 %) use more than one type of CAM. This is similar to western studies of CAM use in CVD patients [23, 25], but not well studied in Singapore. This could be due to the variability between studies in grouping types of CAM. The use of multiple CAM is significant as it increases the probability of adverse medication side effects [22].

Understanding the factors that influence a patient to use CAM is important in helping the physician discuss such therapies with the patient. External influences (78.1 %) form the greatest “push factor” for CAM use, of which friends and family (62.5 %) was most common reason cited for its use. This is congruent with other studies done in Singapore [7, 9, 12, 26]. A survey of 704 CAM users in Singapore listed recommendation by friends and family (70.6 %) as the top reason for their CAM use [26]. Family tradition was found to be an important reason for the usage of CAM in a general study of CAM use in Singapore [7]. Another Singapore study of cancer patients found that friends, other cancer patients and family were their main source of information of CAM [9]. In contrast, in Western cohorts, internal influences, namely adverse drug reactions of conventional therapy [27], perceived proven benefit of CAM [24, 28] and few side effects of CAM [24, 28] were the main drivers for CAM use [29]. This could be due to Asian cultural differences, in which opinions of the family are greatly valued in the choice of medical treatment [30].

Studies using Western populations have found CAM users to be more likely female, middle aged, affluent and well-educated [4, 31]. In our study, these factors were not found to be associated with CAM use, highlighting the possible effect of cultural and environmental differences. In our study, Malay ethnicity was significantly associated with less CAM use than Chinese in CVD patients. In several Singapore studies on general CAM users [7], cancer patients [8, 9] and epilepsy [12], Chinese ethnicity was found to be associated with CAM use. A possible explanation is the prevalent use of TCMs for the treatment of CVD risk factors (eg. HTN and DM) and cardiovascular diseases in the Chinese [32].

In patients with CVD, compliance to conventional western medicine may be affected by CAM use. In a study of hypertensive medication adherence, females CAM users were found to have lower compliance than those who did not use CAM [33]. Our study also showed that CAM users were less compliant to conventional medicine as well as follow up appointments compared to non-CAM users. This has strong implications in the management of patients. Non-compliance to medications and visits has been shown to be a strong predictor of adverse outcomes in CVD patients [18–20]. Addressing a patient’s concerns and expectations about both CAM and the conventional medications may help improve such compliance. In our study, we found that the prevalence of CAM disclosure to physicians is generally low. Similarly, a study done in Singapore showed that 74 % of CAM users did not discuss their use with doctors [7]. This is also shown in a study of caregivers of a paediatric epilepsy population [12]. In that study, up to 80 % of caregivers did not inform their doctors about CAM use because they were not asked to. Western studies also made similar observations for CAM users in general [34] and for CVD [15, 23, 29]. The most cited reason for non-disclosure in our study is that the doctors did not ask them about their usage of CAM (60.5 %). Reasons such as perceived disapproval from doctors [29, 35] and insufficient consult time featured less commonly. This is in line with other studies that also identified the lack of physician input as an important factor [17, 29]. A study [10] on CAM use in cancer patients identified that healthcare professionals perceive themselves to have inadequate knowledge (58.8 %) or were not up to date with the best evidence on CAM use (79.2 %) in oncology. This lack of knowledge may be one of the reasons behind a physicians’ avoidance of raising the CAM issue with patients. This issue has also been highlighted in a study of CAM use in paediatric epilepsy caregivers [12], whereby majority of the healthcare providers did not comment on CAM use when asked by patients. Of note, 72.8 % of our sample cohort was keen to know more about CAM from their doctors. This highlights the role of doctors in providing such information and recommendations about CAM [7]. An open physician-patient setting whereby such issues of CAM can be readily discussed could help improve the therapeutic relationship and potentially improve compliance and possibly outcomes [36].

## Limitations

The study population was from the outpatient cardiovascular clinics at a single tertiary cardiac centre and might not be representative of the general population of patients with CVD. Males were over-represented (73.2 %) in our study, possibly reflecting their higher CVD burden [37]. The impact of CAM usage on CVD outcomes was not assessed in this cross-sectional survey, however the results raise interesting questions for future work.

## Conclusion

The usage of CAM is prevalent amongst our patients with CVD. The use of CAM was associated with poorer reported compliance to medications and consults. Understanding the factors influencing CAM use amongst CVD patients provides medical professionals with an opportunity to better discuss CAM use and potentially enhance the patient-physician interaction.

## Additional file

Additional file 1: Sample of Questionnaire. (DOC 2477 kb)

## Abbreviations

ACE: Angiotensin converting enzyme
ARB: Angiotensin receptor blocker
CAM: Complementary and alternative medicine
CVD: Cardiovascular disease
SPSS: Statistical package for the social sciences
TCM: Traditional Chinese medicine
