# Supplementary figures and images for: Complementary and alternative medicine use amongst patients with cardiovascular disease in Singapore
Source: BMC Complement Altern Med. 2016 Nov 8;16:446. doi: 10.1186/s12906-016-1430-4 (PMC5101719; doi:10.1186/s12906-016-1430-4)

**Additional file 1**

| 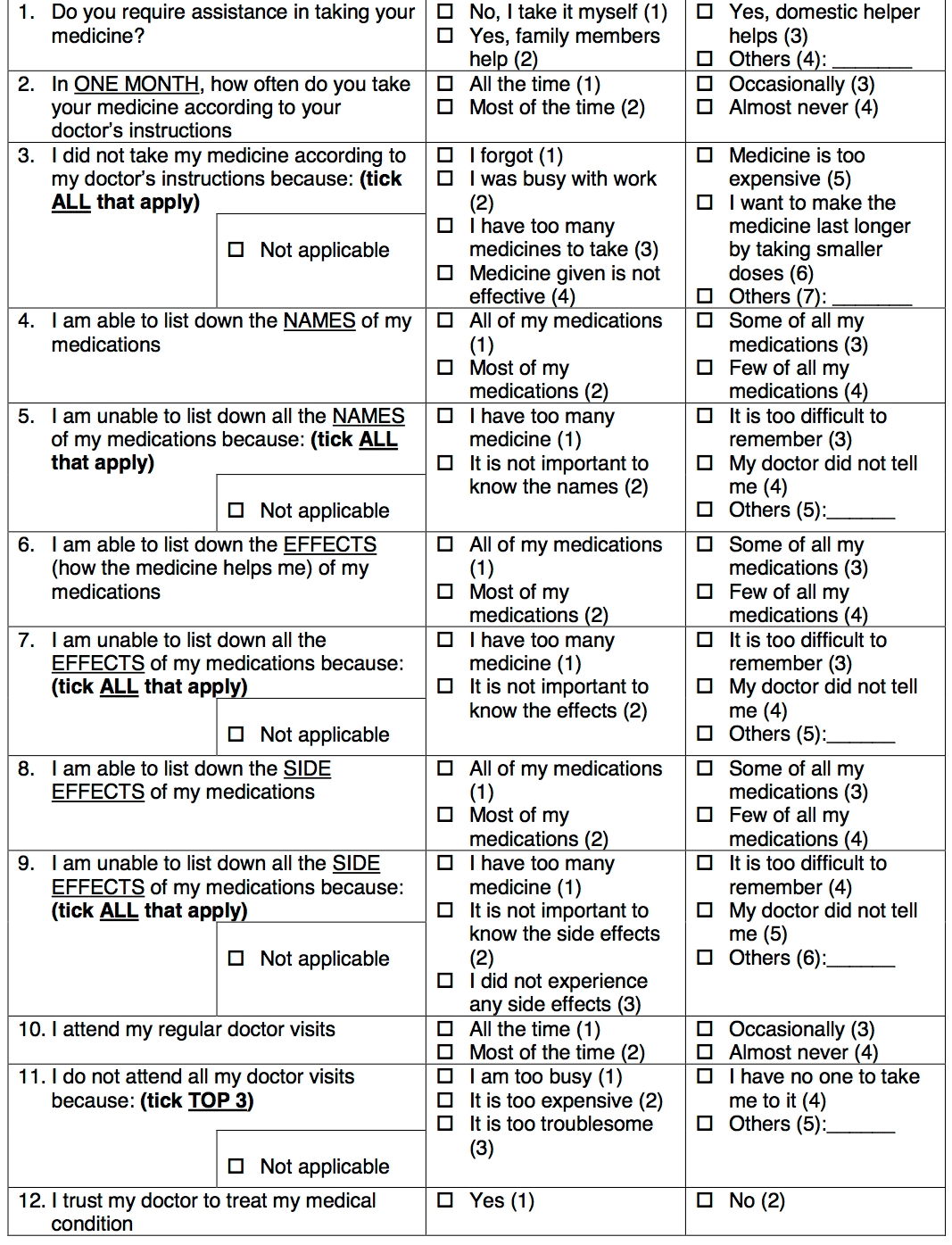 |
| --- |
| 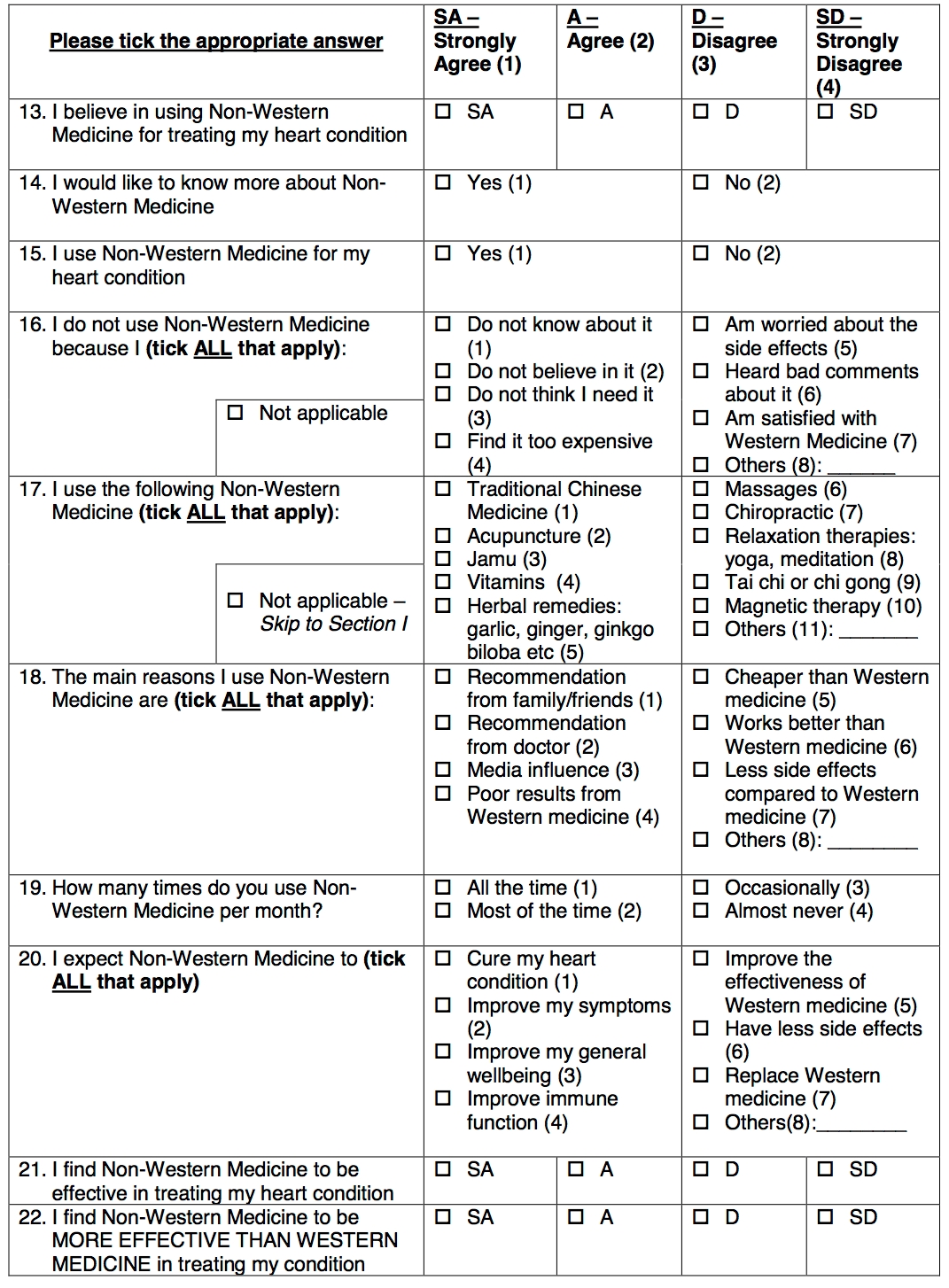 |
| 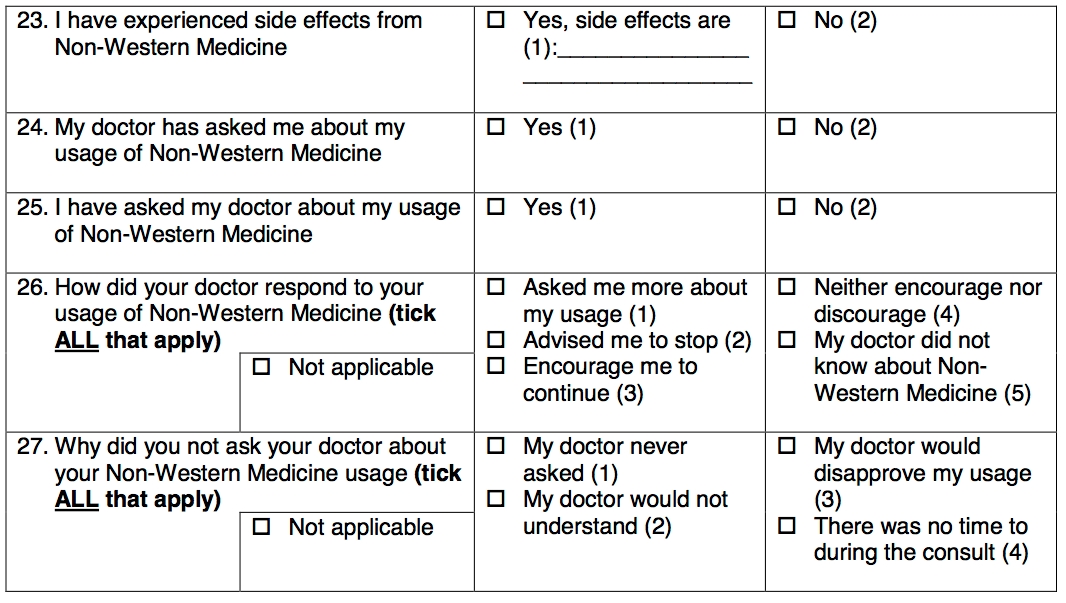 |

Supplement: Additional file 1: — Sample of Questionnaire. (DOC 2477 kb) [file 12906_2016_1430_MOESM1_ESM.doc]
